# Nimesulide-Associated Generalized Bullous Fixed Drug Eruption: A Rare Pharmacovigilance Case Report

**DOI:** 10.7759/cureus.101371

**Published:** 2026-01-12

**Authors:** Mannu Marshal, Shubham Aggarwal, Girish Joseph, Neena Bhatti, Dinesh K Badyal

**Affiliations:** 1 Pharmacology, Christian Medical College and Hospital, Ludhiana, IND

**Keywords:** adverse drug reaction, fixed drug eruption, generalized bullous fixed drug eruption (gbfde), national pharmacovigilance programme, nimesulide, nsaid delayed skin reactions

## Abstract

Generalized Bullous Fixed Drug Eruption (GBFDE) is a severe variant of Fixed Drug Eruption (FDE) characterized by widespread dusky erythematous plaques and blistering, often mimicking Stevens-Johnson Syndrome/Toxic Epidermal Necrolysis (SJS/TEN). Early distinction is critical, as misdiagnosis can lead to delayed or inappropriate management. We report a case of nimesulide-induced GBFDE in a 32-year-old male who developed rapidly progressing dusky plaques and flaccid bullae within 24 hours of self-medicating with an over-the-counter Nimesulide formulation. A prior similar episode following exposure to the same drug strongly supported the diagnosis. Prompt withdrawal of the drug, systemic corticosteroids, and supportive care led to rapid clinical improvement. This case highlights the diagnostic challenges, the dangers of over-the-counter NSAIDs (Non-Steroidal Anti-Inflammatory Drugs) use, and the importance of pharmacovigilance reporting, given that only three cases of nimesulide-associated GBFDE have been documented to date. Increased awareness among clinicians is essential to prevent morbidity and recognize this rare but potentially life-threatening adverse reaction.

## Introduction

Generalized Bullous Fixed Drug Eruption (GBFDE) is a severe Fixed Drug Eruption (FDE) variant defined by typical lesions alongside blisters and erosions. The diagnosis requires involvement of lesions across at least three of six defined anatomical sites. The anatomical sites are head/neck, trunk, back, upper/lower extremities, and genitalia. Due to its widespread nature, dusky skin discoloration, and significant skin detachment, GBFDE is a critical diagnosis often mistaken for Stevens-Johnson Syndrome/Toxic Epidermal Necrolysis (SJS/TEN). Immediate clinical distinction and intensive care are vital because GBFDE is not always benign and carries a rare but significant mortality risk, similar to SJS/TEN [1].

The lesions exhibit dark purplish-red coloration with skin detachment where the epidermis separates from the dermis. Because of its extensive distribution and severe blistering, GBFDE represents a serious dermatologic reaction [2]. The main point of difference between GBFDE and SJS/TEN is GBFDE presents with a shorter latency period and has less severe and minimal mucosal involvement. On the other hand SJS/TEN has a longer latency period and more mucosal involvement [3]. SJS involves <10% BSA (Body Surface Area) which is characterized by mucocutaneous tenderness, erythema, hemorrhagic erosions and epidermal detachment presenting as blisters and areas of denuded skin [4]. In contrast, GBFDE typically shows absent or limited mucosal involvement ranging from 10-50% [2]. However, both of the conditions are associated with significant mortality and need to be identified and managed quickly [2,4].

FDE can affect individuals of all ages, from children to the elderly; however, it is seen most frequently in young to middle-aged adults, with median ages reported between 35 and 60 years [2]. GBFDE represents a rare variant of FDE and accounts for only a small proportion of its cases. Reliable estimates of its true incidence remain unavailable, as there are no large-scale prospective studies specifically evaluating the occurrence of GBFDE [5]. The most common drugs causing GBFDE are nonsteroidal anti-inflammatory drugs (NSAIDs), antibiotics such as cephalosporins and fluoroquinolones, antiepileptic agents, and other medications, including metformin. In addition, several over-the-counter drugs are potential triggers for GBFDE [6].

Nimesulide is a commonly prescribed NSAID belonging to the sulfonamide class, approved for acute pain management, symptomatic treatment of osteoarthritis, and primary dysmenorrhea in adults and adolescents over 12 years of age. Its therapeutic profile, comprising anti-inflammatory, analgesic, and antipyretic effects, is primarily attributed to its selective inhibition of cyclooxygenase-2 (COX-2) [7]. The drug exerts its pharmacological action through preferential COX-2 inhibition and suppression of prostaglandin synthesis, alongside reducing the release of key inflammatory mediators such as cytokines, histamine, neutrophil-derived toxic factors, and cartilage-degrading enzymes [8].

Nimesulide is rapidly distributed and demonstrates extensive albumin binding, with a terminal elimination half-life of approximately four hours. Excretion of the unchanged drug is minimal; instead, nimesulide undergoes hepatic metabolism predominantly via cytochrome P450 to produce its major active metabolite, 4-hydroxynimesulide (M1), which exhibits pharmacological activity similar to the parent compound. Metabolites are mainly excreted through urine (≈70%) and feces (≈30%)[8,9]. The maximum daily dose of nimesulide is 200 mg and it is used to treat chronic and acute pain. If taken chronically for rheumatic diseases, then dose adjustment is done [9].

Nimesulide is generally well tolerated; however, it may cause adverse effects such as headache, dizziness, somnolence, gastrointestinal disturbances including nausea, abdominal discomfort, diarrhea, peripheral edema, and hypersensitivity reactions. Like other NSAIDs, it carries the risk of rare but serious complications, including gastrointestinal ulceration, bleeding, and perforation, increased cardiovascular events such as myocardial infarction and stroke, renal impairment, worsening of asthma, potential effects on fertility and fetal development, and severe acute hypersensitivity reactions such as anaphylaxis, exfoliative dermatitis, and Stevens-Johnson syndrome [10]. Although adverse reactions associated with nimesulide are relatively common, with skin and subcutaneous tissue disorders accounting for approximately 28% of all spontaneous adverse-drug reaction reports in the VigiAccess database, the occurrence of GBFDE remains exceedingly rare. To date, only three cases of GBFDE linked to nimesulide have been documented [11].

This discrepancy between the high proportion of skin-related ADRs and the extremely low number of GBFDE reports underscores the significance of each new case: reporting additional instances of GBFDE is vital to better characterize the true risk, contribute to pharmacovigilance data, and guide clinicians about a potentially life-threatening but under-recognized complication. By presenting this new case, we aim to add to the limited global literature on nimesulide-associated GBFDE, promote awareness among prescribers, and highlight the need for careful drug history, prompt recognition, and avoidance of re-exposure.

## Case presentation

A 32-year-old male presented with a two-day history of fever, for which he had self-medicated with an over-the-counter combination of nimesulide 100 mg tablet twice daily. Within 24 hours of initiating the medication, he developed diffuse erythematous-to-dusky macules and patches on the trunk and extremities, associated with a significant burning sensation. The lesions initially appeared over the medial aspect of the right arm and rapidly progressed to involve bilateral upper limbs and thighs, with some lesions demonstrating areas of central duskiness suggestive of evolving necrosis. Over the next day, the patient developed multiple flaccid bullae of varying sizes over previously involved sites. Some bullae ruptured spontaneously, leaving behind yellowish crusted erosions. There was no mucosal involvement (oral, ocular, or genital) and no systemic symptoms apart from the cutaneous manifestations.

**Figure 1:**
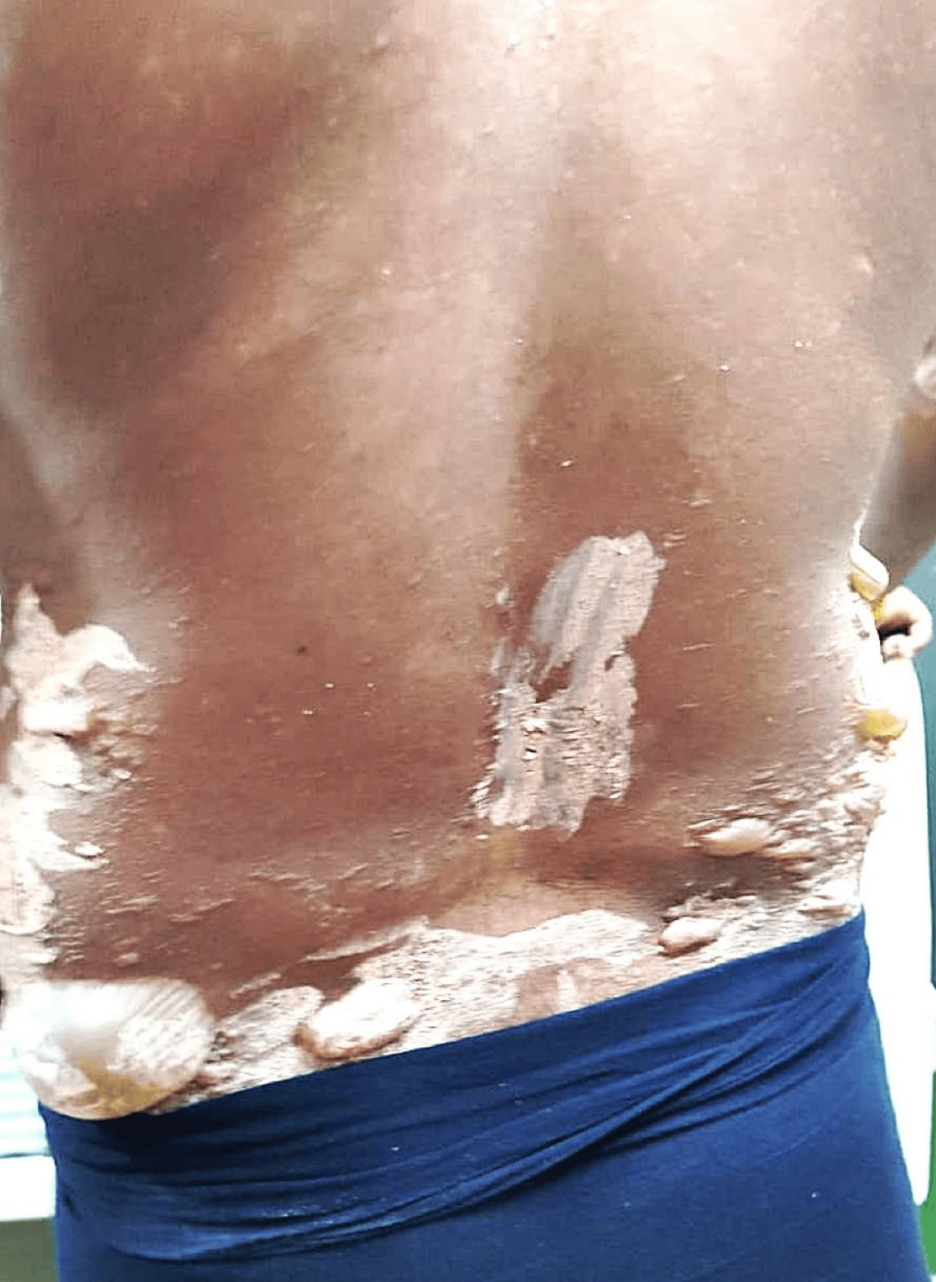
Generalized bullous fixed drug eruption involving the trunk Diffuse erythematous to dusky hyperpigmented plaques over the trunk with multiple flaccid bullae and areas of epidermal detachment. Some bullae have ruptured, resulting in erosions with crusting. The distribution and morphology are consistent with generalized bullous fixed drug eruption.

The patient was evaluated in the dermatology outpatient department, and the diagnosis of GBFDE was made based on the acute onset of recurrent, sharply demarcated dusky plaques with flaccid bullae following re-exposure to the same culprit drug, the absence of mucosal involvement, and a previous similar reaction to Nimesulide. This is shown in Figure 1.

**Figure 2:**
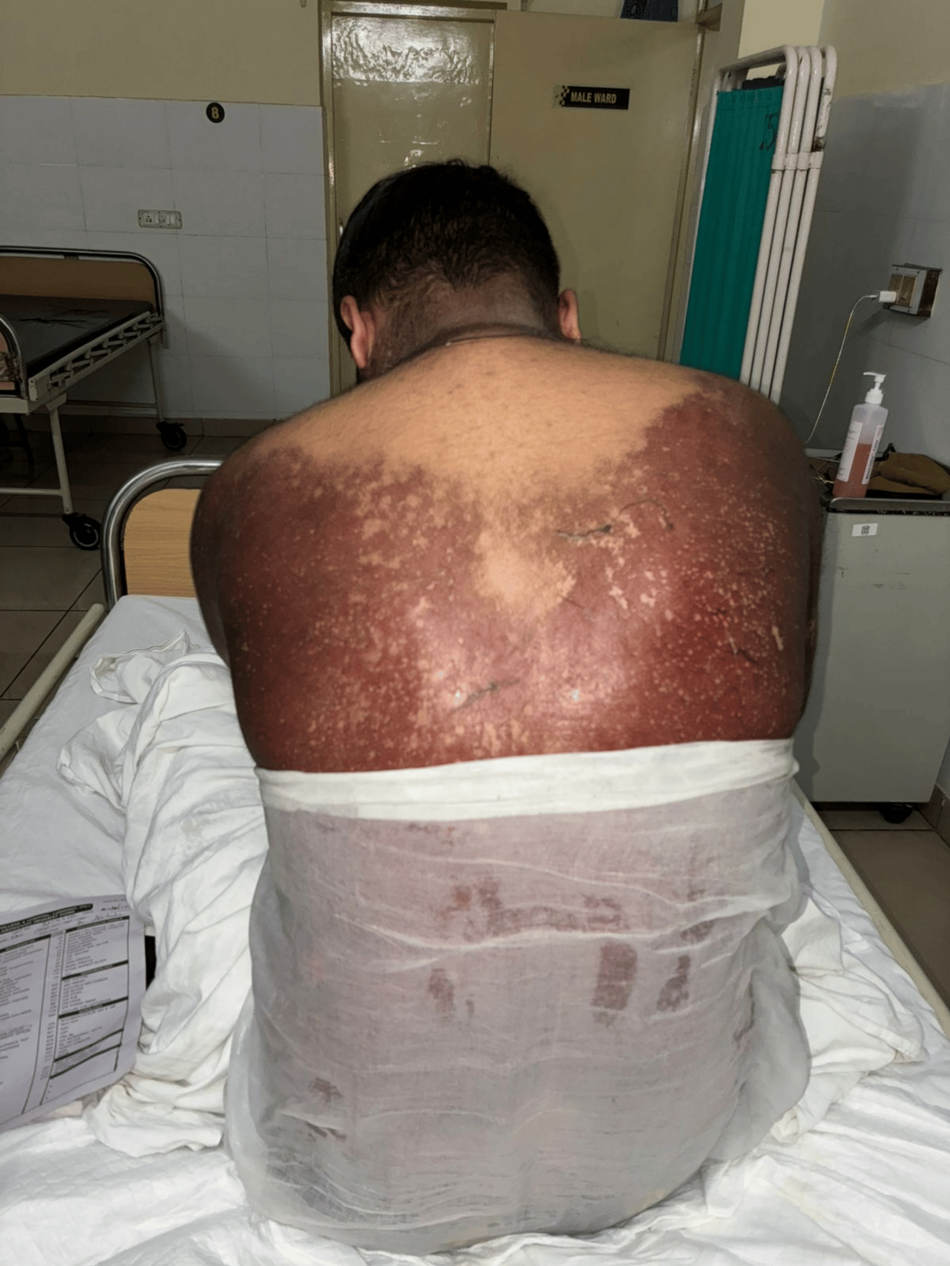
Post-recovery hyperpigmentation following generalized bullous fixed drug eruption Diffuse residual hyperpigmentation over the upper back and trunk following resolution of generalized bullous fixed drug eruption after withdrawal of the offending drug and supportive treatment. No active bullae, erosions, or epidermal detachment are visible, indicating clinical recovery

The clinical pattern and temporal relationship strongly favored GBFDE over other severe cutaneous adverse reactions such as SJS/TEN. There was no history of other drug exposures, food allergy, autoimmune disease, or recent infection. The suspected offending drug was immediately discontinued, and the patient was managed with systemic corticosteroids, including hydrocortisone 100 mg intravenously stat, followed by a single high dose of prednisolone 200 mg. Symptomatic relief for pruritus was provided with hydroxyzine 25 mg at bedtime. Intravenous clindamycin 600 mg every eight hours was administered to prevent secondary bacterial infection. Supportive wound care was given with sterile Jelonet dressings and regular emollient application to maintain skin hydration and promote re-epithelialization. The patient showed significant improvement within a few days, with resolution of erythema and no further development of new lesions. He was discharged in stable condition with counselling to strictly avoid nimesulide and structurally related NSAIDs in the future. The post-discharge condition of the patient is shown in Figure 2.

The causality assessment using the Naranjo Adverse Drug Reaction Probability Scale yielded a score of 7, indicating a probable association between the reaction and the nimesulide-containing medication. The Naranjo probability scale comprises 10 questions, each answered with “yes,” “no,” or “don’t know,” and assigned a score of −1, 0, +1, or +2 depending on the response. Based on the total score, causality is categorized as definite (≥9), probable (5-8), possible (1-4), or doubtful (≤0). Overall scores can range from −4 to +13 [12]. If we consider the severity of the reaction, the Modified Hartwig scale classified the ADR as moderate (Grade 4), as the reaction required hospitalization, withdrawal of the offending drug, and active therapeutic intervention [13].

A detailed summary of the ADR assessment is provided in Table 1, where causality was assessed using the Naranjo Adverse Drug Reaction Probability Scale, severity was graded according to the Modified Hartwig and Siegel Severity Assessment Scale, and preventability was evaluated using the Schumock and Thornton criteria [13,14].

**Table 1:** Summary of adverse drug reaction assessment using standardized pharmacovigilance tools

Assessment tool	Criteria applied	Result in this case	Interpretation
Naranjo Adverse Drug Reaction Probability Scale	Temporal relationship, improvement on drug withdrawal, recurrence on re-exposure, exclusion of alternative causes	Score = 7	Probable adverse drug reaction
Modified Hartwig and Siegel Severity Assessment Scale	Drug withdrawal required, hospitalization needed, active medical management required, no permanent disability or death	Level 4	Moderate severity adverse drug reaction
Schumock and Thornton Preventability Scale	Prior history of reaction, availability of safer alternatives, self-medication with over-the-counter drug	Yes	Probably preventable adverse drug reaction

This case was reported to the AMC (Adverse Drug Monitoring Centre), under the Pharmacovigilance Programme of India (PvPI) at Christian Medical College & Hospital, Ludhiana. Written informed consent was obtained from the patient for publication of this case and accompanying images.

## Discussion

The World Health Organization (WHO) defines an adverse drug reaction (ADR) as "a response to a medication that is noxious and unintended and occurs at doses normally used in man" [15]. ADRs can significantly affect a patient's quality of life and contribute to increased morbidity, mortality, and healthcare costs worldwide [16]. GBFDE causes pain, pruritus, flare-ups, and post-inflammatory hyperpigmentation, which greatly impacts the quality of life [17]. In our patient, the rapid onset of diffuse dusky-to-erythematous patches progressing to flaccid bullae, with recurrence on re-exposure to Nimesulide, absence of mucosal involvement, and prompt resolution on withdrawal strongly support the diagnosis of GBFDE.

FDE is one of the most common cutaneous adverse drug reactions (CADR); however, the GBFDE remains a rare entity. A similar case reported in 2023 described GBFDE mimicking epidermal necrolysis, highlighting the frequency with which GBFDE is mistaken for more serious cutaneous adverse reactions (SCARs) such as Toxic Epidermal Necrolysis (TEN) or Stevens-Johnson syndrome (SJS) [18]. Similarly, a recent observational study of CADRs at a tertiary centre in 2024 revealed that CADRs considerably lower patients' quality of life, particularly when there is widespread skin involvement or symptoms like burning or itching. Higher Dermatology Life Quality Index (DLQI) scores, indicating poorer quality of life, were associated with widespread lesions, with FDE as a prevalent presentation [19].

The possibility of such serious responses may be underestimated due to the growing use of NSAIDs, frequently as over-the-counter fixed-dose combos, and regular self-medication in many areas. In fact, previous case reports have shown that nimesulide causes bullous FDE and GBFDE [20,21]. The standard concept of sensitization and reactivation mediated by resident memory T-cells in the skin is supported by our patient's history of a prior reaction to the identical drug combination three years before, albeit probably less severe, followed by a more dramatic, widespread reaction upon re-exposure. The underlying mechanism is believed to be a delayed type IVc hypersensitivity reaction, which is postulated to damage the skin's basal layer and activate resident CD8+ T cells as a result. This reaction results in the release of several mediators, including interferon (IFN)-γ, and the recruitment of additional immune cells, which damage keratinocytes and melanocytes and eventually cause the distinctive lesions to emerge [1].

It has been observed that GBFDE and SJS/TEN can look similar; a few clinical differences can help tell them apart. GBFDE more commonly affects older adults, who often appear less systemically unwell compared with patients with SJS/TEN. Another helpful clue is timing. GBFDE almost always appears quickly after taking the offending drug, usually within 48 hours, and always within one to two weeks. In contrast, SJS/TEN tends to develop later, most often one to three weeks after exposure. The rash patterns also differ: SJS/TEN lesions often merge together and may form atypical target-like spots, while GBFDE lesions stay more sharply defined with areas of normal skin between them. Healing also differs-GBFDE usually resolves with only darkened skin, while SJS/TEN frequently leaves significant scarring, especially on mucosal surfaces [2].

The cornerstone of management in this case was withdrawal of the suspect product and providing supportive therapy such as systemic corticosteroids, topical care, and prevention of secondary infection [21]. Recent reports highlighting severe and occasionally fatal cases of GBFDE have prompted interest in cyclosporine as a therapeutic option. To date, six cases treated with cyclosporine have been published, five in adults and one in a child. Adults received 3-5 mg/kg/day for 5-14 days, with rapid resolution of erythema and cessation of blister formation. The pediatric patient improved within 24 hours on an initial dose of 5 mg/kg/day divided twice daily, tapered to 2.5 mg/kg/day over the following two weeks [2]. Our patient's favorable result and comparatively speedy recovery were probably made possible by intensive early intervention.

Under-recognition of GBFDE, particularly by non-dermatologists, is concerning, given the widespread use of NSAIDs in various communities. In addition to increasing clinician knowledge and adding to pharmacovigilance databases, publishing well-documented examples may encourage more cautious prescribing or the avoidance of needless fixed-dose combinations. The risks of repeated exposures and self-medication are highlighted in the case of our patient by the previous lesser reaction followed by a more severe episode.

Limitations

This case report has certain limitations inherent to single-patient observations. As an isolated case, the findings cannot be generalized, and broader epidemiological trends or drug-specific risk estimates cannot be established. The report is also limited by the absence of long-term follow-up, preventing assessment of recurrence patterns or delayed complications. Additionally, the diagnosis relied primarily on clinical features and temporal association, which, although strong, may not capture the full spectrum of GBFDE presentations. Finally, because pharmacovigilance reporting is known to be incomplete, the true incidence of Nimesulide-associated GBFDE may be underestimated, limiting the ability to contextualize this case within larger population data.

Future directions

Future work should focus on developing multicenter prospective registries to systematically document GBFDE cases, enabling better characterization of incidence, drug associations, clinical outcomes, and recurrence patterns. Incorporating standardized patient-reported outcome measures, including DLQI (Dermatology Life Quality Index) or other dermatology-specific QoL (Quality of Life) tools, would improve understanding of the psychosocial and functional impact of GBFDE. Further studies exploring immune pathways, particularly the role of resident memory T-cells in recurrence and severity, may offer insights into targeted therapies. Emerging treatments such as cyclosporine warrant further evaluation through controlled clinical studies, especially for severe or rapidly progressive cases. Finally, public health strategies aimed at reducing inappropriate over-the-counter NSAID use and strengthening pharmacovigilance reporting may help prevent avoidable drug-related cutaneous reactions.

## Conclusions

GBFDE is a rare but severe cutaneous adverse reaction that requires prompt recognition and differentiation from SJS/TEN due to diagnostic and therapeutic implications. This case demonstrates the potentially life-threatening consequences of nimesulide exposure, particularly in settings where self-medication with NSAIDs is common. Early drug withdrawal and supportive management resulted in rapid improvement. Given the extremely limited number of nimesulide-associated GBFDE cases reported worldwide, this case provides valuable evidence for the existing literature and underscores the need for heightened clinician awareness, patient education, and robust pharmacovigilance. Continued reporting and research are essential to improving diagnosis, prevention, and long-term outcomes for patients with GBFDE.

## References

[1] Makris M, Papapostolou N, Koumprentziotis IA, Pappa G, Katoulis AC (2024). Nimesulide-induced fixed drug eruption followed by etoricoxib-induced fixed drug eruption: an unusual case report and review of the literature. J Clin Med.

[2] Anderson HJ, Lee JB (2021). A review of fixed drug eruption with a special focus on generalized bullous fixed drug eruption. Medicina (Kaunas).

[3] Mitre V, Applebaum DS, Albahrani Y, Hsu S (2017). Generalized bullous fixed drug eruption imitating toxic epidermal necrolysis: a case report and literature review. Dermatol Online J.

[4] Joseph G, Bhatti N, Badyal DK, Kaur P (2025). Stevens-Johnson syndrome: an adverse drug reaction with various drugs. Indian J Physiol Pharmacol.

[5] Yeh JW, Liu WT (2025). Fixed drug eruption-A retrospective review of 146 patients in a tertiary hospital in Southern Taiwan. Dermatol Sin.

[6] Shrestha P, Stone CA Jr, Phillips EJ (2025). Fixed drug eruption and generalized bullous fixed drug eruption: Insights from an analysis of the FDA Adverse Event Reporting System. J Allergy Clin Immunol Pract.

[7] Wei W, Evseenko VI, Khvostov MV (2021). lubility, permeability, anti-inflammatory action and in vivo pharmacokinetic properties of several mechanochemically obtained pharmaceutical solid dispersions of nimesulide. Molecules.

[8] Tiwaskar M, Charde S, Balankhe N (2025). Nimesulide: critical appraisal of safety and efficacy in acute pain. J Assoc Physicians India.

[9] Sun X, Xue KL, Jiao XY, Chen Q, Xu L, Zheng H, Ding YF (2016). Simultaneous determination of nimesulide and its four possible metabolites in human plasma by LC-MS/MS and its application in a study of pharmacokinetics. J Chromatogr B Analyt Technol Biomed Life Sci.

[10] LiverTox: Clinical and Research Information on Drug-Induced Liver Injury. https://www.ncbi.nlm.nih.gov/books/NBK547948/.

[11] (2025). VigiAccess. WHO global database of individual case safety reports. Uppsala: Uppsala Monitoring Centre.

[12] Naranjo CA, Busto U, Sellers EM (1981). A method for estimating the probability of adverse drug reactions. Clin Pharmacol Ther.

[13] Hartwig SC, Siegel J, Schneider PJ (1992). Preventability and severity assessment in reporting adverse drug reactions. Am J Hosp Pharm.

[14] Pirmohamed M, James S, Meakin S (2004). Adverse drug reactions as cause of admission to hospital: prospective analysis of 18 820 patients. BMJ.

[15] Abhilasha P, Bhatti N, Joseph G, Badyal DK (2024). Sodium valproate-induced hyperammonemia: a case series in a tertiary care hospital. Cureus.

[16] Kaur M, Mongia AK, Joseph G, Bhatti N, Badyal DK (2025). Ondansetron-induced dystonia: an uncommon case report. Natl J Pharmacol Ther.

[17] Shaker G, Mehendale T, De La Rosa C (2022). Fixed drug eruption: an underrecognized cutaneous manifestation of a drug reaction in the primary care setting. Cureus.

[18] Paulmann M, Reinkemeier F, Lehnhardt M, Mockenhaupt M (2023). Case report: generalized bullous fixed drug eruption mimicking epidermal necrolysis. Front Med (Lausanne).

[19] Shivani Shivani, Sinha R, Arya AK, Jaykar KC, Pallavi UK (2025). Cutaneous adverse drug reactions and their impact on the quality of life of patients: a study at a tertiary care centre. IMC J Med Sci.

[20] Malheiro D, Cadinha S, Rodrigues J, Vaz M, Castel-Branco MG (2005). Nimesulide-induced fixed drug eruption. Allergol Immunopathol (Madr).

[21] Barootes HC, Peebles ER, Matsui D, Rieder M, Abuzgaia A, Mohammed JA (2021). Severe generalized bullous fixed drug eruption treated with cyclosporine: a case report and literature review. Case Rep Dermatol.

